# Exercise modulates central and peripheral inflammatory responses and ameliorates methamphetamine-induced anxiety-like symptoms in mice

**DOI:** 10.3389/fnmol.2022.955799

**Published:** 2022-08-29

**Authors:** Guo-Fen Re, Hong Li, Ji-Qun Yang, Yue Li, Zunyue Zhang, Xiaocong Wu, Ruiyi Zhou, Deshenyue Kong, Huayou Luo, Yi-Qun Kuang, Kun-Hua Wang

**Affiliations:** ^1^School of Medicine, Yunnan University, Kunming, China; ^2^National Health Commission (NHC) Key Laboratory of Drug Addiction Medicine, Kunming Medical University, Kunming, China; ^3^Yunnan Narcotics Control Bureau, Kunming, China; ^4^The Third People’s Hospital of Kunming, Kunming, China; ^5^Scientific Research Laboratory Center, First Affiliated Hospital of Kunming Medical University, Kunming, China; ^6^Department of Gastrointestinal and Hernia Surgery, First Affiliated Hospital of Kunming Medical University, Kunming, China

**Keywords:** methamphetamine, acute withdrawal, cytokine, anxiety-like symptoms, treadmill exercise, microglia, neuroinflammation

## Abstract

Anxiety-like symptoms are common symptoms of methamphetamine (METH) users, especially in the acute withdrawal period, which is an important factor for the high relapse rate during METH acute withdrawal. Exercise has been demonstrated to relieve anxiety-like symptoms during METH withdrawal, but the underlying mechanisms of this anti-anxiety effect are still unclear. Activated microglia and abnormal neuroinflammation play an important role in the pathogenesis of anxiety-like symptoms after METH withdrawal. Moreover, peripheral immune factors were also significantly associated with anxiety symptoms. However, the effects of treadmill exercise on microglial function and neuroinflammation in the striatum and hippocampus during acute METH withdrawal have not been reported. In the current study, we found severe peripheral immune dysfunction in METH users during acute withdrawal, which may in part contribute to anxiety symptoms during METH acute withdrawal. We also showed that 2 weeks of METH exposure induced anxiety-like symptoms in the acute withdrawal period. Additionally, METH exposure resulted in increased microglial activation and proinflammatory cytokines released in the mouse striatum and hippocampus during acute withdrawal. We next evaluated the effects of treadmill exercise in countering anxiety-like symptoms induced by METH acute withdrawal. The results showed that anxiety-like symptoms induced by acute METH withdrawal were attenuated by coadministration of treadmill exercise. In addition, treadmill exercise counteracted METH-induced microglial activation in the mouse striatum and various subregions of the hippocampus. Furthermore, treadmill exercise also reversed the increase in proinflammatory cytokines induced by acute METH withdrawal in the mouse striatum, hippocampus and serum. Our findings suggest that the anti-anxiety effect of treadmill exercise may be mediated by reducing microglial activation and regulating central and peripheral inflammatory responses.

## Introduction

Methamphetamine (METH) is a highly addictive central stimulant that can damage the central nervous system, resulting in hallucinations, anxiety, depression, cognitive impairment, and other mental disorders, as well as serious physical harm, including chronic poisoning, weight loss, infectious diseases, liver and kidney dysfunction, and cardiovascular and cerebrovascular diseases (Grelotti et al., 2010; Jones et al., 2020; Han et al., 2021). According to the 2021 World Drug Report (UNODC, 2021), 0.5% of the global population aged 15–64 years had used amphetamine-type stimulants (ATS) in 2019. In addition, more than 95% of ATS laboratories discovered between 2015 and 2019 were used to make methamphetamine, and the drug accounted for 72% of ATS seized during the same period; in East and Southeast Asia, crystalline methamphetamine is mainly used in many countries. In China, among the 2.2 million drug users officially registered in 2019, synthetic drugs (mainly methamphetamine) accounted for 55% (Office of China National Narcotics Control Commission, Drug Situation in China, 2019). METH is highly addictive, and its synthetic precursors, such as ephedrine and pseudoephedrine, are easy to obtain, resulting in a high proportion of METH users, causing serious health hazards and affecting economic development and social harmony (UNODC, 2021). Therefore, it is necessary to explore effective methods to reduce METH addiction and relapse. The strong neuroexcitatory effect of METH, intense craving and refractory mental symptoms after withdrawal lead to a high relapse rate in METH users, which is the focus and difficulty of METH abstinence and treatment.

Previous clinical studies have shown that METH users often experience psychiatric symptoms, and there was a dose-dependent increase in the incidence of psychotic symptoms among METH users (McKetin et al., 2006, 2013). Anxiety is one of the most prominent symptoms in the withdrawal period of METH, especially in the acute withdrawal stage (Zhao et al., 2021), and its incidence is related to the duration of METH exposure (Zorick et al., 2010). Furthermore, a study of a large sample of methamphetamine-dependent subjects found that 30.2% of METH users had anxiety disorders induced by METH (Salo et al., 2011). Anxiety symptoms during the METH withdrawal period are associated with a high relapse rate and poor prognosis (Hellem, 2016), and a large sample clinical trial of METH users showed that after 3 years of treatment, METH users with anxiety symptoms have poorer drug use outcomes and increased health service consumption (Glasner-Edwards et al., 2010). Therefore, the treatment of anxiety-like symptoms in the METH acute withdrawal period is an important measure to reduce craving and relapse. Currently, there is no specific drug treatment for METH use disorder. Clinically, antipsychotics are widely used to treat METH addiction, such as mirtazapine, but the therapeutic effect is limited and has great side effects (Shoptaw et al., 2009; Coffin et al., 2020). In addition, psychosocial interventions are effective for METH use disorders, and a study suggested that Group Music Therapy is an effective treatment method to enhance treatment motivation for female METH use disorder (Wu et al., 2020). In recent years, brain physical stimulation therapy has shown great potential in the treatment of drug addiction and drug withdrawal symptoms (Zhu et al., 2016; Liang et al., 2018). A randomized, double-blind clinical trial showed that repetitive transcranial magnetic stimulation targeting the left dorsolateral prefrontal cortex (DLPFC) could improve METH withdrawal symptoms (Liang et al., 2018), which requires larger clinical trials for verification. Increasing studies have found that exercise is an effective therapy to treat drug use disorders (Linke and Ussher, 2015; He et al., 2021), and moderate-intensity exercise may be the most promising optional intensity to promote mental health (Paolucci et al., 2018). A randomized population experiment suggested that a structured exercise program could effectively improve the anxiety and depression symptoms of METH withdrawal (Rawson et al., 2015). In addition, some clinical and preclinical studies have also proven that reasonable exercise can effectively alleviate mental health disorders of METH withdrawal, such as anxiety and depression (Barbour et al., 2007; Carneiro et al., 2015; Huang et al., 2019). However, the neurobiological mechanism of exercise detoxification is still unclear. Previous studies have found that exercise can improve nerve damage caused by METH exposure by regulating neurochemical balance (dopamine and brain-derived neurotrophic factor), improving oxidative stress, restoring the integrity of the blood–brain barrier and promoting the generation of neurons and glia, thus improving addiction and relapse (Morais et al., 2018). However, the study of exercise modulating central and peripheral inflammatory responses to improve anxiety symptoms during METH withdrawal has not been reported.

Methamphetamine can lead to systemic multisystem dysfunction, especially in the nervous and immune systems, which is closely related to METH addiction, relapse and withdrawal symptoms. Previous clinical studies found that peripheral immune factors are related to neuropsychiatric disorders induced by METH. For example, inflammatory cytokine/chemokine levels were found to be associated with anxiety, depression and memory impairment in METH users, and the immune factors CRP (C-reaction protein), IL-8, MMP-3, SCF, VEGF, eotaxin-1 and IL-23 were significantly correlated with the anxiety symptoms induced by METH (Huckans et al., 2015). Moreover, serum IL-2R, IL-6, IL-8, and IL-10 levels were associated with the severity of mental symptoms in METH use disorders (Yang X. et al., 2020). In addition, many animal experiments have also proven that METH can induce central and peripheral inflammatory responses, damage the blood–brain barrier and aggravate neurotoxicity, which may be the pathological basis of anxiety-like symptoms induced by METH (Prakash et al., 2017). This shows that immune dysfunction plays an important role in mediating nerve injury and psychosis induced by METH. Therefore, modulating central and peripheral immune disorders is of great value to improve the anxiety-like symptoms induced by METH.

The striatum is a component of the extrapyramidal system and mainly receives fibers from the frontal cortex and thalamus. An increasing number of studies have illustrated that the striatum is involved in controlling anxiety-like behavior (Joling et al., 2018; Dias et al., 2019), and the specific biological mechanism needs to be further studied. In addition, the hippocampus is also involved in the regulation of emotional states, including anxiety (Bannerman et al., 2014). Furthermore, the prefrontal cortex and amygdala also functionally contribute to the control of anxiety (Salzman and Fusi, 2010). Microglia are central innate immune cells that mediate the central inflammatory response. Numerous lines of evidence that central microglia are involved in the pathogenesis of neuropsychiatric disorders such as anxiety and depression (Yang and Zhou, 2019). New evidence shows that microglia will change from a relatively resting state to a variety of activated states during the development of regional specialization, and these activated states are closely related to the different functions of microglia in these different brain regions and different microenvironments (Li et al., 2022). Moreover, when microglia are activated by various pathological conditions, they will produce various phenotypes of microglia, which depends on the background and type of stressors or pathology (Wolf et al., 2017). Under stress and pathological conditions, microglia are activated and migrate to the focal area, producing inflammatory responses and affecting synaptic pruning and other neural structures (Terry et al., 1991; Kreisel et al., 2014; Yirmiya et al., 2015). This is associated with psychosis and the development of neurodegenerative diseases (Al Mamun et al., 2020). METH can activate microglia, induce neuroinflammation and lead to nerve damage (Sekine et al., 2008; Dang et al., 2021), which may be the pathological basis of anxiety-like symptoms during METH withdrawal. Increasing studies have shown that CD68 is an ideal marker for the activation and phagocytosis of microglia (Murphy et al., 2005). Microglia coexpressed with ionized calcium-binding adapter molecule 1 (Iba-1) and CD68 were activated microglia. Exercise has beneficial effects on many systems of the body. Some research has shown that regular exercise can counteract persistently low levels of peripheral and central inflammation (Collao et al., 2020; Scheffer and Latini, 2020). In addition, exercise enhances memory and inhibits brain inflammation via plasma clusterin (De Miguel et al., 2021).

Here, we first analyzed the cytokine levels of METH users in the acute withdrawal period compared with healthy people. The clinical results indicated that the peripheral immune response is seriously maladjusted, which may be an important reason for anxiety-like symptoms in METH users during the acute withdrawal period. Next, we constructed a mouse model of acute METH withdrawal–induced anxiety-like symptoms and measured the metabolism of the model mice with a metabolic cage. Then, we investigated the beneficial effects of moderate-intensity treadmill exercise on acute METH withdrawal–induced anxiety-like symptoms concerning its ability to reduce microglial activation and neuroinflammation in the mouse hippocampus and striatum. We also examined the levels of cytokines in the serum of model mice, and the results suggested that exercise can reduce the peripheral inflammatory response in mice with acute METH withdrawal. Moderate-intensity treadmill exercise improves acute METH withdrawal–induced anxiety-like symptoms that may occur through modulating the peripheral inflammatory response and reducing microglial activation and neuroinflammation in the mouse striatum and hippocampus.

## Materials and methods

### Study participants and sample collection

The METH users were treated in the Yunnan Drug Rehabilitation Center under strict management. All METH participants provided informed consent and signed written informed consent before being included in the study. The study participants were males with HIV(−), HBV(−), and HCV(−) and without major diseases, such as cardiovascular diseases. These participants included 23 healthy controls and 22 METH users in the acute withdrawal period. The included healthy controls were matched with the life background of METH users. The basic information of all participants is shown in Table 1. The types of drugs used by participants were determined by urine tests. The recruitment procedures and study methods described were approved by the Research Ethics Committee of the First Affiliated Hospital of Kunming Medical University (No. 2018 Lunshen L42) and performed according to the relevant regulations. The blood samples of all participants were collected at 08:00–10:00 AM using vacuum blood collection tubes containing EDTA anticoagulant and then centrifuged at 3000 rpm for 15 min. Then, the upper layer of plasma was transferred to another Eppendorf tube and stored at −80°C for testing.

**TABLE 1:** The basic information and cytokine levels (pg/ml) of study participants.

	Control	METH (AW)	*P*-value
Gender (Male/Female)	23/0	22/0	NA
Withdrawal days	NA	9.684 ± 5.292	NA
Age (years)	32.26 ± 11.37	34.36 ± 6.94	0.4604
Ethnicity			0.1650
Han	5 (21.7%)	9 (40.9%)	
Minority	18 (78.3)	13 (59.1%)	
**Cytokines (pg/ml)**			
TNF-α	73.70 ± 13.84	105.4 ± 41.46	**0.0012**
IL-6	1.20 ± 0.55	10.69 ± 8.75	**<0.0001**
IL-1β	8.21 ± 1.95	1.66 ± 2.53	**<0.0001**
IL-2	7.90 ± 2.95	5.83 ± 3.15	**0.0280**
IL-12p70	2.42 ± 0.64	5.41 ± 3.14	**<0.0001**
IL-7	72.57 ± 28.08	148.6 ± 63.29	**<0.0001**
IL-13	3.83 ± 3.11	6.95 ± 5.35	**0.0007**
IL-15	327.4 ± 219.3	149.5 ± 130.9	**0.0046**
IL-9	983.6 ± 130.4	361.8 ± 271.8	**<0.0001**
IL-1ra	388.8 ± 171.8	1095 ± 1098	**<0.0001**
RANTES	2170 ± 5152	79129 ± 67494	**<0.0001**
Eotaxin/CCL11	165.6 ± 86.29	609.8 ± 301.8	**<0.0001**
IP-10	2680 ± 730.9	7057 ± 6340	**0.0002**
MIP-1α	5.32 ± 3.15	2.97 ± 1.61	**0.0044**
MIP-1β	290.3 ± 45.75	258.1 ± 111.3	**0.0112**
Basic-FGF	336.8 ± 100.4	95.68 ± 68.56	**<0.0001**
G-CSF	290.8 ± 77.99	428.7 ± 316.9	**0.0492**
PDGF-BB	15181 ± 8380	9623 ± 14150	**0.0087**

The data were expressed as mean ± standard deviation (SD). AW, acute withdrawal. The age and cytokines were analyzed by the unpaired t-test or Mann Whitney test. The ethnicity was analyzed by the Chi-square test. p < 0.05 was considered as a significant difference. The bold values represent a statistically significant value.

### Animals and ethical statement

Male C57BL/6J mice (11 weeks old and weighing 23 ± 2 g) were provided by SBF Biotechnology Co., Ltd. (Beijing, China). The mice were housed under a 12-h light/dark cycle (light on at 7:00, off at 19:00) at an appropriate temperature (21 ± 2°C) and were free to food and water. The mice were randomly assigned. Before the experiment began, all mice were allowed to adapt to the housing environment for 1 week. During the experiment, each group of mice was treated without bias. All of the experimental procedures in this study were approved by the Animal Experiment Ethics Review Committee of Kunming Medical University (Approval number: kmmu20211261) and performed according to the National Research Council’s Guide for the Care and Use of Laboratory Animals.

### Experimental design and drug management

**FIGURE 1:**
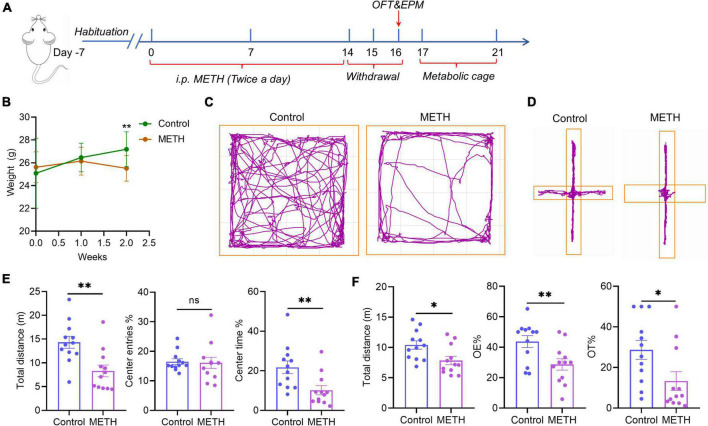
Acute METH withdrawal–induced anxiety-like symptoms. **(A)** The timeline of the mouse model of anxiety-like symptoms induced by METH acute withdrawal. **(B)** The weight changes that occurred in mice during 2 weeks of METH exposure. **(C)** The behavioral trajectories of mice in the METH group and control group in the open field test. **(D)** The behavioral trajectories of mice in the METH group and control group in the elevated plus maze experiment. **(E)** The behavioral experiment results of the OFT test in the METH group (*n* = 12) and control group (*n* = 12). **(F)** The behavioral experiment results of the EPM test in the METH group (*n* = 12) and control group (*n* = 12). The data were compared with an unpaired *t*-test and are expressed as the mean ± SEM. Significance is shown as *p < 0.05, **p < 0.01, ***p < 0.001, and ****p < 0.0001.

The establishment of a mouse model of anxiety-like symptoms induced by acute METH withdrawal (Figure 1): METH exposure was achieved by intraperitoneal injection of METH dissolved in sterile saline twice a day for 14 constitutive days, with a stepwise increase of 0.2 mg/kg per injection, and the interval between two injections per day was 4 h (first injection time: 13:00, second injection: 17:00). Each mouse was injected with 0.1 ml each time, the initial concentration was 2 mg/kg, and the last dose concentration was 7.4 mg/kg. This METH dose regimen was adjusted with reference to previous studies (Liskiewicz et al., 2019; Ghavimi et al., 2021), and the increasing dosing is in line with the habits of drug users. Mice in the control group were injected with saline in the same way. After METH withdrawal for 48 h (acute METH withdrawal), the open field test (OFT) and elevated plus maze (EPM) behavioral experiments were carried out, and metabolic cage monitoring was carried out on the 17th to 21st days.

### Treadmill running scheme

Twelve-week-old C57BL/6J male mice were randomly divided into four groups (*n* = 8–10 per group) (Figure 3): (1) controls treated with saline, (2) controls treated with saline and moderate-intensity treadmill exercise, (3) METH exposure, and (4) METH exposure and moderate-intensity treadmill exercise. METH exposure was administered as shown above. The moderate-intensity treadmill exercise program was modified from a mouse exercise protocol used in related studies (Qu et al., 2020; Cheng et al., 2021). Treadmill exercise was carried out on a small animal treadmill (SA101B, SANS, China). The treadmill exercise lasted for 3 weeks, and the first week was adaptive training (10 m/min, 0° slope, 20 min on day 1, 40 min on day 2, 60 min/day on days 3 to 6, rest on day 7). The next 2 weeks were formal training (10 m/min, 0° slope, 60 min/day, 6 days/week) and co-administered METH. The time of treadmill exercise was from 8:00 to 9:00, and METH was injected intraperitoneally 4 h after treadmill exercise (first injection time: 13:00, second injection: 17:00). Behavioral experiments in the OFT and EPM were conducted 48 h after METH withdrawal (Figure 3A). Following the behavioral tests, the mice were sacrificed to collect samples. Five mice in each group were randomly selected for cardiac perfusion and brain tissue sectioning. The other mice were sacrificed by cervical dislocation after taking blood from the eyeballs of the mice. The striatum and hippocampus were dissected on ice, immediately immersed in liquid nitrogen, and then stored at –80°C until the cytokine assay.

### Drugs

Methamphetamine drugs were supplied by the Narcotics Department of Yunnan Provincial Public Security Administration, and the methamphetamine was dissolved in 0.9% sterile saline for intraperitoneal injection.

### Metabolic cage

The High-Resolution Behavioral and Metabolic Phenotyping System for Mice (Sable Systems International, America) is mainly used to monitor the respiration, metabolism and behavior of mice. The system can measure the respiratory entropy, gas changes (oxygen, carbon dioxide, and water vapor), food intake, water intake, exercise and energy consumption of eight mice in real time and synchronously. In this study, 48 h after METH withdrawal (Figure 1A), eight mice (including four mice in the control group and four mice in the METH group) were placed in eight metabolic cages simultaneously for metabolic monitoring. All mice were weighed before and after the metabolic cage test, and metabolic cage monitoring lasted for 5 days. Finally, the metabolic data in the middle 3 days were selected for analysis using CalR (Mina et al., 2018), and the body weight data used for analysis were the average value of body weight before and after metabolic monitoring. One mouse in the METH group was excluded from the data analysis because of significant outliers.

### Open field test

The open field test (OFT) is the most widely used behavioral method to detect locomotor activity and anxiety-like behaviors in mice (Seibenhener and Wooten, 2015). The experimenters assessed the anxiety-like behavior of mice by the time they stayed in the central area and the number of times they entered the central area. All mice were allowed to adapt to the test room for at least 3 h in advance of the experiment, and the room was kept dim and quiet during the experiment. In short, mice were placed in an open field box and allowed to explore independently for 5 min. After each trial, the box floor was cleaned with 75% ethanol to clear the smell of the mouse to avoid giving odor cues to subsequent mice. The movement trajectory and movement parameters of the mice were recorded by an infrared video camera and analyzed by ANY-maze Video Tracking Software (Stoelting Co., United States). The evaluated parameters from the open field test included the total movement distance, the number of times entering the central area and the residence time of the central area.

### Elevated plus maze test

The elevated plus maze test (EPM) is one of the most commonly used tests for measuring anxiety-like behavior (Komada et al., 2008). The test was based on the mice’s natural aversion to open and elevated areas, as well as their natural exploration behavior in a new environment. The device comprises an open arm and a closed arm that cross in the middle; their intersection forms the central area. The mice could access all arms and move freely between them. The number of times to enter the open arm and the time spent in the open arm were used as indicators of anxiety in mice. All mice were allowed to adapt to the test room for at least 3 h in advance of the experiment, and the room was kept dim and quiet during the experiment. Briefly, the experimenter needs to hold the mouse with its back to the experimenter, place it at the junction of the open arm and closed arm, and let the mouse face the open arm, allowing them to explore independently for 5 min. After each trial, the device floor was cleaned with 75% ethanol to clear the smell of the mouse to avoid giving odor cues to subsequent mice. The movement parameters of the mice were recorded with the same equipment as the open field test. The evaluated parameters from the test included the percentage of open arm entry times and dwell time.

### Perfusion and tissue processing

After the behavioral test, five mice were randomly selected from each group used for cardiac perfusion and brain extraction. The mice were anesthetized with 8% chloral hydrate (6 ml/kg, intraperitoneal injection) and transcardially perfused with precooled saline (30 ml) followed by 4% paraformaldehyde (30 ml). Then, the cerebrum of each mouse was removed. The brain tissue was postfixed in 4% paraformaldehyde at 4°C for 24 h and then immersed in sucrose solution (dissolved in PBS) with an increased concentration gradient to dehydrate (20 and 30% sucrose solutions were used for 24 h, respectively). The brain tissue was cut into 30 μm serial sections using a frozen slicer (CM1950, Leica, Germany). At least 12 brain slices were cut continuously in each brain region and stored at –80°C.

### Immunofluorescence

Among the 12 serial sections of each sample, the sections were randomly selected for the immunofluorescence experiment. The slices were placed in a moisturizing box at room temperature for 30 min, and then free-floating brain sections were rinsed two times with PBS for 5 min each time. The sections were incubated with 1x Quick Antigen Retrieval Solution for Frozen Sections (pH = 7.4, abs9207, Absin, Shanghai, China) at room temperature for 5 min and then rinsed with PBS 3 times for 5 min each time. A circle was drawn around the brain tissue with the immunohistochemical pen, and then the sections were blocked with blocking buffer at room temperature for 60 min. They were then incubated overnight in a mixture of two primary antibodies: goat polyclonal IgG anti-Iba-1 (dilution at 1:500, NB100-1028, Novus Biologicals, United States) and rabbit monoclonal IgG anti-CD68 (dilution at 1:800, #97778, Cell Signaling Technology, MA, United States). The rabbit anti-GFAP (dilution at 1:500, ab207165, Abcam) was incubated overnight. The two primary antibodies were diluted with antibody diluent. The next day, the sections were washed three times with PBS for 5 min each time and incubated with fluorophore-conjugated secondary antibodies at a dilution of 1: 800 (diluted with antibody diluent) for 80 min at room temperature. The secondary antibodies were Alexa Fluor^®^ 594-conjugated AffiniPure donkey anti-goat†† IgG (H + L) and Alexa Fluor^®^ 488-conjugated AffiniPure donkey anti-rabbit IgG (H + L). The Alexa Fluor^®^ 647 Rabbit monoclonal to NeuN was incubated for 2 h. Subsequently, the sections were washed three times with PBS, and the anti-fluorescent quench sealing agent (including DAPI) (abs9235, Shanghai, China) was used for nuclear staining and sealing. Fluorescent images were captured with a confocal microscope (N-SIM/C2si, Nikon, Japan). At least five representative images of each brain subregion were acquired from each sample. The Iba-1^+^ cells and Iba-1^+^/CD68^+^ cells in each image were manually counted. The percentage of activated microglia was expressed as the ratio of the number of Iba-1^+^/CD68^+^ cells to Iba-1^+^ cells.

### Cytokine assays

The levels of 27 cytokines in the plasma of all participants were detected by Luminex technology (Luminex-X-200, Bio-Rad, United States) according to the manufacturer’s guidelines. The Bio-Plex Pro Human Cytokine 27-plex Assay Kit (M500KCAF0Y, 64103331, Bio-Rad) could detect 27 cytokines, including IFN-γ, IL-1ra, IL-2, IL-4, IL-5, IL-6, IL-7, IL-8, IL-9, IL-12 (p70), IL-13, IL-15, IL-17A, TNF-α, IL-1β, IL-10, IP-10, basic FGF, eotaxin/CCL11, G-CSF, GM-CSF, MCP-1 (MCAF), MIP-1α/CCL3, MIP-1β/CCL4, PDGF-BB, RANTES/CCL5 and VEGF. Detailed plasma cytokine detection and calculation methods have been described in our previous studies (Zhang et al., 2021).

The concentrations of cytokines in mouse striatum, hippocampus and serum samples were tested using Luminex detection technology (Luminex-X-200, Bio-Rad, United States) in accordance with the manufacturer’s guidelines. The mouse classic inflammatory factor combination Kit (LXR-MultiDTM-10, LabEx, Shanghai, China) could detect these cytokines, including IL-10, IL-12p70, CXCL1, IFN-γ, TNF-α, IL-1β, IL-2, IL-4, IL-5, and IL-6. The blood of mice was collected from eyeballs with a 1.5 ml centrifuge tube without anticoagulant, stood at 4°C for 2 h, and then centrifuged for 20 min (4°C, 3000 × *g*). The supernatant was taken and stored at –80°C for detection. The mouse serum was diluted twice before Luminex detection. The brain samples were homogenized with an appropriate amount of lysate (abs9225, Absin, Shanghai, China) containing protease inhibitor (abs9161, Absin, Shanghai, China) and centrifuged at 13,000 rpm for 10 min at 4°C. The tissue supernatant was collected and stored at −80°C, and the total protein concentration was detected by the BCA method (P0010, Beyotime, Shanghai, China). The cytokine levels were detected by the Luminex technique. Standard curves were generated to determine the unknown sample concentration. Because some cytokine levels in brain regions and serum samples were lower than the lower limit of detection, only cytokine indices that were detected in at least three samples were included in the data analysis.

### Data and statistical analysis

Statistical analysis and plotting were performed using GraphPad Prism 8.0 (GraphPad Software, San Diego, CA, United States). The data of the metabolic cage were also analyzed with CalR Version 1.3 (CalR, Mouse Metabolic Phenotyping Center Harvard Digestive Disease Center) (Mina et al., 2018), and the generalized linear model (GLM) was used to infer the effects of body mass and activity on metabolic variables in mice. The age of the population is expressed as the mean ± standard deviation (SD). The unpaired t test was used for age comparison, and the chi-square test was used for ethnic comparison. All animal experimental data are expressed as the mean ± standard error (SEM). Continuous variable data were evaluated for normality and homogeneity of variance. The independent two groups of data were compared with an unpaired *t* test or Mann–Whitney test. The parametric data were analyzed by one-way analysis of variance (ANOVA) for multiple comparisons followed by a *post hoc* Tukey test, and the two-way ANOVA for four groups comparison followed by Tukey’s test. The non-parametric data were analyzed by the Kruskal–Wallis test followed by a *post hoc* Dunn’s multiple comparison test. All statistical tests were two-tailed, and *p* < 0.05 was considered a significant difference.

## Results

### The basic information and alterations of plasma cytokine profile of study participants

The study recruited 22 male METH users at withdrawal for 7–14 days (AW, acute withdrawal) and 23 healthy volunteers matched to the life background of METH users. The basic information of the participants is shown in Table 1. There were no significant differences in age or ethnicity between the METH group and the control group. Among the results of 27 cytokines, IFN-γ and GM-CSF were excluded from the data analysis because more than 50% of the samples were lower than the lower quantitative range. The results revealed that the levels of the proinflammatory cytokines TNF-α, IL-6, IL-12p70 and IL-7 were significantly elevated in METH users compared with healthy controls (Table 1), indicating that there is a significant inflammatory response in the acute withdrawal period of METH. In particular, compared with the control group, the level of the anti-inflammatory cytokine IL-1ra was also markedly increased in the METH group, which could block the proinflammatory effect of IL-1. Moreover, compared to the control group, the levels of the cytokines IL-13, RANTES/CCL5, eotaxin/CCL1, IP-10 and G-CSF prominently increased in METH users (Table 1). However, the levels of the cytokines IL-1β, IL-2, IL-15, IL-9, MIP-1α/CCL3, MIP-1β/CCL4, basic FGF and PDGF-BB were significantly decreased in METH users compared with the control group (Table 1). The above results illustrated that there is obvious peripheral immune dysfunction after acute withdrawal of METH. It cannot be ignored that the levels of the cytokines IL-4, IL-5, IL-8, IL-10, IL-17A, VEGF and MCP-1 (MCAF) were not significantly different between the two groups (Supplementary Table S1).

### Methamphetamine induced anxiety-like symptoms during acute withdrawal and affected energy metabolism in mice

The time axis of the METH-induced withdrawal anxiety-like symptoms model is shown in Figure 1A. The weight of mice in the control group increased gradually over time, while the weight of mice in the METH group was significantly lower than that in the control group after 2 weeks (*p* = 0.0018) (Figure 1B). Figures 1C,D show the trajectories of mice in the OFT and EPM experiments, respectively. The total distance in the OFT showed that mice with METH withdrawal traveled a significantly less distance than control mice (*p* = 0.0025) (Figure 1E), indicating that mice in the METH group have less spontaneous activity and prefer to stay in the corner. Moreover, the percentage of center time in the METH group were significantly lower than those in the control group (*p* = 0.0080), however, the percentage of center entries had no significant difference between the METH group and control group (Figure 1E). The EPM test showed that the percentage of open arm residence time and open arm entries in the METH group were significantly lower than those in the control group (*p* = 0.0284 and *p* = 0.0109, respectively) (Figure 1F). The above two experiments showed that METH exposure could induce anxiety-like symptoms in acute withdrawal.

**FIGURE 2:**
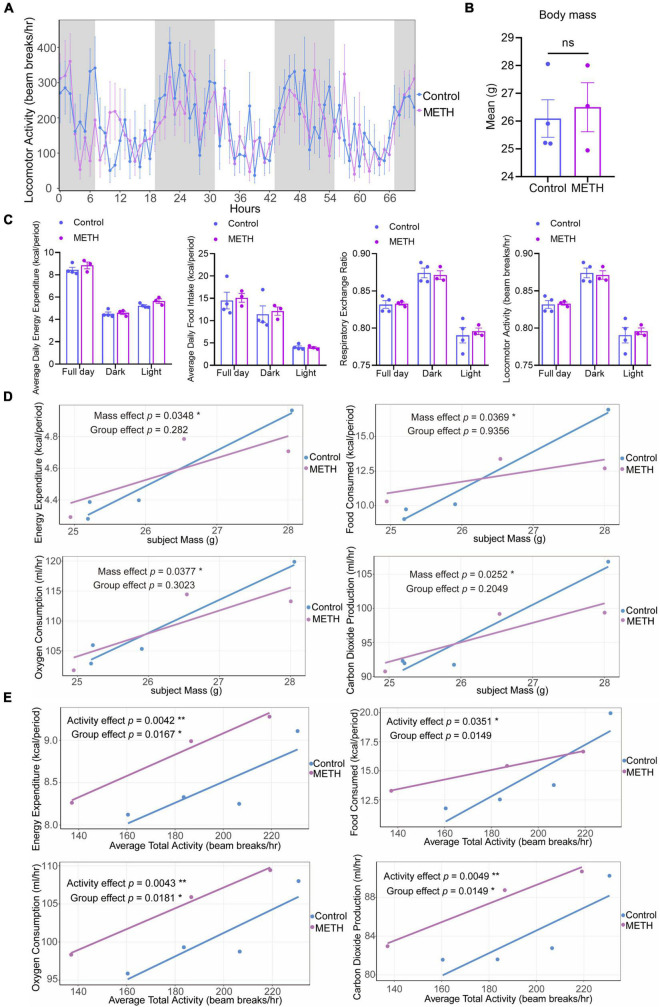
Methamphetamine (METH) exposure resulted in an imbalance of energy metabolism in mice. **(A)** The circadian locomotor activity in a metabolic cage of mice in the METH group and control group within 3 days. The gray shadows represent the dark (start at 19:00), and white represents the light (start at 7:00). **(B)** The average body mass of mice in the two groups before and after the metabolic cage test. **(C)** Changes in metabolic parameters (average daily energy expenditure, average daily food intake, respiratory exchange ratio, and locomotor activity) in the METH group (*n* = 3) and control group (*n* = 4). **(D)** GLM-based regression plots in which the average of the selected metabolic variables is plotted against body mass. **(E)** GLM-based regression plots in which the average of the selected metabolic variables is plotted against the average total activity. The data in **(B,C)** were compared with an unpaired *t* test and are expressed as the mean ± SEM. The generalized linear model was used to analyze the effects of body mass and activity on metabolic variables in mice of the METH group and control group. Significance is shown as *p < 0.05, **p < 0.01, ***p < 0.001, and ****p < 0.0001.

In the metabolic cage results, Figure 2A shows the locomotor activity of mice over three days, and the gray shadow represents the night (19:00-07:00), indicating that mice are more active at night (Figure 2A). There was no significant difference in average body mass between the METH group and the control group mice for metabolic analysis (Figure 2B). These metabolic variables, including average daily energy expenditure, average daily food intake, respiratory exchange ratio and locomotor activity, were not significantly different between the METH group and the control group (Figure 2C). In addition, the average food intake, respiratory exchange ratio and locomotor activity in all mice were higher during the dark than during the light (Figure 2C).

**FIGURE 3:**
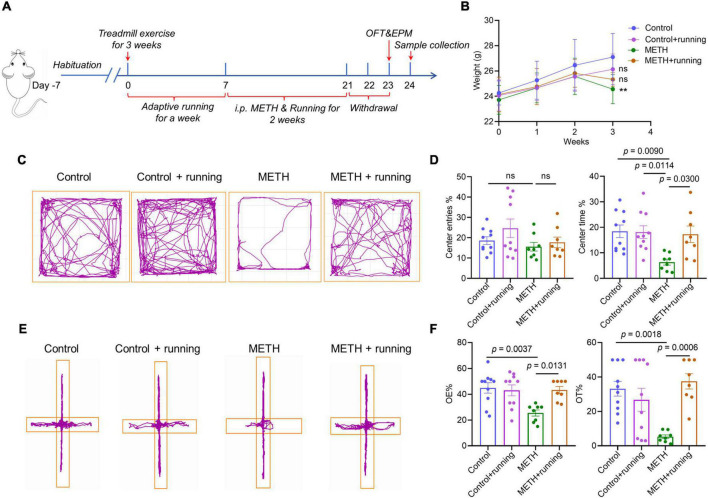
Treadmill exercise improved the acute METH withdrawal–induced anxiety-like symptoms in mice. **(A)** The timeline of the mouse model of treadmill exercise ameliorating anxiety-like symptoms in the METH acute withdrawal period. **(B)** The changes in body weight in four groups of mice within 3 weeks, and the data were analyzed by one-way analysis of variance (ANOVA) for multiple comparisons followed by a *post hoc* Tukey test. **(C)** The behavioral trajectories of mice in the four groups in the open field test. **(E)** The behavioral trajectories of mice in the four groups in the elevated plus maze experiment. **(D)** The behavioral experiment results of the OFT test in the control group (*n* = 10), control + running group (*n* = 10), METH group (*n* = 8), and METH + running group (*n* = 8). **(F)** The behavioral experiment results of the EPM test in the control group (*n* = 10), control + running group (*n* = 10), METH group (*n* = 8), and METH + running group (*n* = 8). The data are expressed as the mean ± SEM, and *p* < 0.05 was considered a significant difference.

Figures 2D,E show GLM-based regression plots (Figures 2D,E). For each subject, the average of the selected metabolic variables is plotted against the selected mass and total activity variables. The mass effect was significant for these metabolic variables (*p* < 0.05), but the group effect showed no significant difference after accounting for body mass (Figure 2D), indicating that body mass could significantly affect the energy expenditure, food consumption, oxygen consumption, and carbon dioxide production of mice. When controlling the body mass of mice, there was no significant difference in metabolic parameters between the METH group and the control group. In addition, the activity effect was significant for these metabolic variables (*p* < 0.05) (Figure 2E), and the group effect also showed a significant difference for energy expenditure, oxygen consumption and carbon dioxide production after accounting for activity (*p* < 0.05) (Figure 2E). This illustrates that activity could significantly affect the energy expenditure, food consumption, oxygen consumption and carbon dioxide production of mice. When the activity of mice was the same, the energy consumption and oxygen and carbon dioxide production of mice in the METH group were significantly higher than those in the control group, while there was no significant difference in food consumption between the two groups. These results suggest that METH can affect the energy consumption and oxygen and carbon dioxide production of mice after accounting for activity.

In addition, we measured the changes of behavior and some metabolic parameters in mice within 3 h after intraperitoneal injection of METH 4.6 mg/kg (the average concentration of this animal mode) using the High-Resolution Behavioral and Metabolic Phenotyping System for Mice. The results were shown in Supplementary Figure S1. The results showed that during METH exposure, the locomotor activity of mice increased significantly, and the total distance was also significantly higher than the control group, indicating that behavior sensitization occurred in mice during METH exposure. Moreover, compared with the control group, the water consumption of mice decreased significantly during METH exposure. However, within 3 h after intraperitoneal injection of METH, the total food consumption, energy expenditure, respiratory exchange ratio, oxygen consumption and carbon dioxide production of mice were not significant differences compared with the saline injection group (control group).

### Moderate-intensity treadmill exercise improved the acute methamphetamine withdrawal–induced anxiety-like symptoms in mice

At the end of the third week, the mouse weight in the METH group was significantly lower than that in the control group (*p* < 0.05), and the mouse weight in the exercise intervention group was higher than that in the METH group (Figure 3B). The OFT results showed that treadmill exercise significantly increased the percentage of center time of METH-exposed mice (*p* < 0.05) (Figures 3C,D). Furthermore, the EPM experiment also verified the OFT results and showed that moderate-intensity treadmill exercise significantly increased the entries and dwell time in the open arms of METH-exposed mice (*p* < 0.05) (Figures 3E,F). The above results indicated that moderate-intensity treadmill exercise improved the anxiety-like symptoms of mice with acute METH withdrawal.

### Moderate-intensity exercise reduces the peripheral inflammatory response in mice with acute methamphetamine withdrawal

To investigate the effect of treadmill exercise on the serum cytokine levels of METH withdrawal mice, the levels of 10 classical cytokines in the serum of mice were examined with Luminex technology. Because the levels of IL-1β and IL-6 were lower than the lower limit of detection, we included only eight other cytokines for statistical analysis (Table 2). We found that the level of TNF-α was remarkably elevated in the serum of mice in the METH group relative to the control group (*p* < 0.05), which is consistent with the change in the population cytokine TNF-α level in the present study. After treadmill exercise intervention, the level of TNF-α in the serum of mice was significantly reduced (*p* < 0.05) (Table 2). However, the levels of other cytokines, including IFN-γ, IL-2, IL-4 IL-5, IL-10, CXCL1 and IL-12p70, were not significantly different among the three groups (Table 2).

**TABLE 2:** Treadmill exercise regulate the levels of serum cytokines in METH acute withdrawal mice.

Serum cytokines	Control (pg/ml)	METH (pg/ml)	METH + running (pg/ml)	^a^ *P-value*	METH vs. Control *^b^Adj. P-value*	METH + running vs. METH *^b^Adj. P-value*
TNF-α	15.41 ± 3.74	40.33 ± 8.14	16.09 ± 6.16	**0.0166**	**0.0247**	**0.0355**
IFN-γ	5.13 ± 1.84	8.39 ± 3.79	5.20 ± 2.81	0.9336	>0.999	>0.999
IL-2	0.84 ± 0.09	3.63 ± 2.70	9.97 ± 5.76	0.3279	>0.999	>0.999
IL-4	0.75 ± 0.13	0.60 ± 0.07	0.68 ± 0.12	0.6637	>0.999	>0.999
IL-5	3.09 ± 0.67	2.38 ± 0.21	2.87 ± 0.37	0.7364	>0.999	>0.999
IL-10	7.17 ± 0.60	6.70 ± 0.69	6.97 ± 0.91	0.8970	0.8877	0.9653
CXCL1	34.42 ± 2.70	27.85 ± 3.14	41.81 ± 8.41	0.4243	0.6161	0.8821
IL-12p70	148.5 ± 24.45	101.3 ± 24.95	146.7 ± 16.70	0.2780	0.3225	0.3487

^a^Kruskal–Wallis test (the non-parametric data) or Ordinary one-way ANOVA (the parametric data) was used to compare the cytokine levels among control, METH, and METH + running groups.

^b^Dunn’s multiple comparisons test (the non-parametric data) or Tukey’s multiple comparisons test (the parametric data) was used for pairwise comparison between groups. P-value < 0.05 (two-tailed) was considered to indicate statistical significance.

The bold values represent a statistically significant value.

### Moderate-intensity treadmill exercise reduced the ratio of activated microglia in the striatum of mice with acute methamphetamine withdrawal

**FIGURE 4:**
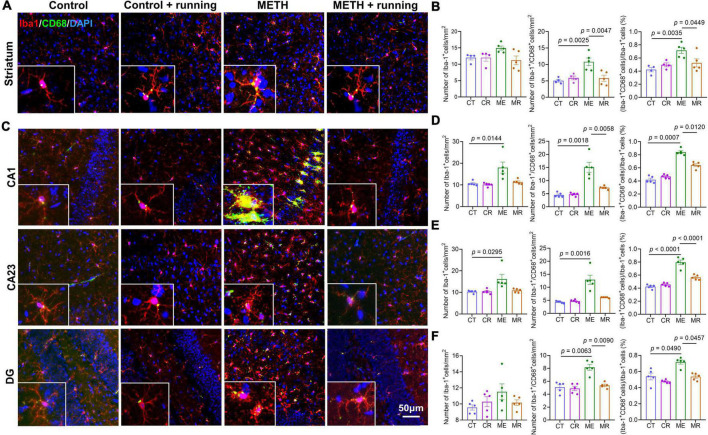
Treadmill exercise decreased microglial activation in the striatum and subregions of hippocampus of mice with acute METH withdrawal. **(A)** Immunofluorescence staining of striatal sections from four groups of mice. The bottom left corner of each picture is the enlarged pictures for the CD68 and Iba-1. **(B)** The number of Iba-1^+^ cells (left), the number of Iba-1^+^/CD68^+^ cells (middle), and the percentage of Iba-1^+^/CD68^+^ microglia in Iba-1^+^ cells (right) from four groups (*n* = 4–5 each group) in the mouse striatum. The data are expressed as the mean ± SEM, and *p* < 0.05 was considered a significant difference. **(C)** Immunofluorescence staining of hippocampal subregion (CA1, CA23, DG) sections from the four groups of mice. The bottom left corner of each picture is the enlarged pictures for the CD68 and Iba-1. **(D–F)** The number of Iba-1^+^ cells (left), the number of Iba-1^+^/CD68^+^ cells (middle), and the percentage of Iba-1^+^/CD68^+^ microglia in Iba-1^+^ cells (right) from four groups (n = 4-5 each group) in the mouse CA1 **(D)**, CA23 **(E)**, and DG **(F)** subregions. The data are expressed as the mean ± SEM, and *p* < 0.05 was considered a significant difference. Iba-1, ionized calcium-binding adapter molecule 1; CT, control; CR, control + running; ME, METH; MR, METH + running.

**FIGURE 5:**
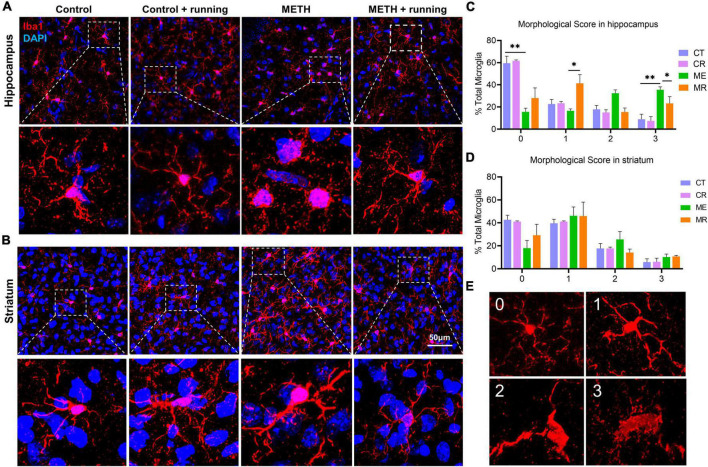
The effects of treadmill exercise on the morphology of microglia in the hippocampus and striatum of mice with acute METH withdrawal. **(A,B)** Representative image of microglia morphology in the hippocampus **(A)** and striatum **(B)**. **(C,D)** These percentages are morphological scores for each type of microglia in the hippocampus **(C)** and striatum **(D)**. **(E)** Morphologically, the microglia with long and thin branching processes scored 0, 1 for microglia with thicker processes with branches; 2 for microglia with thick, retracted processes with few branches; and 3 for round microglia without processes. The data are expressed as the mean ± SEM. Significance is shown as *p < 0.05, ^**^p < 0.01, ^***^p < 0.001, and ^****^p < 0.0001.

To illustrate the effect of treadmill exercise on microglial activation, we showed the coexpression of Iba-1 and CD68 on microglia in the striatum of mice using the immunofluorescence costaining technique. CD68 is a cytoplasmic glycoprotein with a relative molecular weight of 110 000, which is highly expressed in activated and phagocytized microglia. Red fluorescence indicated Iba-1^+^ microglia, green fluorescence indicated CD68^+^ microglia, and blue fluorescence indicated the nucleus (Figures 4A,C). The density of Iba-1^+^/CD68^+^ microglia and the percentage of Iba-1^+^/CD68^+^ in Iba-1^+^ cells in the striatum were significantly different among the four groups [*F*_(3,14)_ = 9.163, *p* = 0.0013; *F*_(3,14)_ = 7.043, *p* = 0.004]. Although there was no significant difference in the number of Iba-1^+^ cells in the striatum, the number of Iba-1^+^ cells in the METH group was higher than that in the other groups (Figure 4B). Specifically, compared with the control group, the density of Iba-1^+^/CD68^+^ microglia and the percentage of Iba-1^+^/CD68^+^ in Iba-1^+^ cells in the striatum in the METH group were prominently increased (*p* = 0.0025 and *p* = 0.0035, respectively) (Figure 4B). In addition, the density of Iba-1^+^/CD68^+^ microglia and the percentage of Iba-1^+^/CD68^+^ in Iba-1^+^ cells in the striatum were markedly decreased in the METH + running group compared with the METH group (*p* = 0.0047 and *p* = 0.0449, respectively) (Figure 4B). However, morphological analysis of microglia showed that there was no significant difference among the four groups (Figures 5B,D). The morphological analysis of Iba1-positive cell was based on the protocol in previous work (Hong et al., 2016). Additionally, we provided a low magnification image (Iba-1^+^/CD68^+^) of the striatum in Supplementary Figure S2. This finding indicated that microglia were activated in the METH acute withdrawal period, which could be significantly improved by moderate-intensity treadmill exercise.

### Moderate-intensity treadmill exercise reduced the ratio of activated microglia in the subregions of the hippocampus of methamphetamine acute withdrawal mice

To explain the beneficial effect of treadmill exercise on microglia in the subregions of the hippocampus, we assessed the number of activated microglia (Iba-1^+^/CD68^+^ cells) and their proportion in Iba-1 cells in the subregions of the hippocampus after treadmill exercise. In the hippocampal CA1 and CA23 regions, relative to the control group, the number of Iba-1^+^ cells in the METH group increased significantly (*p* = 0.0144 and *p* = 0.0295, respectively) (Figures 4D,E). However, there was no difference in the number of Iba-1^+^ cells in the DG region among the four groups (Figure 4F). Moreover, it can be seen from the morphology that the microglia in the METH group are in an obviously activated state, which is characterized by an increase in the cell body, the shortening of the process, and the cell shape being round or rod-shaped (Young and Morrison, 2018; Figure 5A), while there was no obvious activated morphology of microglia in the hippocampus of mice in the METH + running group (Figure 5A). The number of Iba-1^+^/CD68^+^ microglia in the CA1, CA23 and DG and the proportion of Iba-1^+^/CD68^+^ microglia in the Iba-1^+^ cells in CA1, CA23 and DG were significantly different among the four groups. In particular, compared with the control group, the number of Iba-1^+^/CD68^+^ microglia and proportion of Iba-1^+^/CD68^+^ microglia in the Iba-1^+^ cells in the CA1, CA23 and DG subregions were markedly elevated in the METH group (*p* = 0.0018, *p* = 0.0016 and *p* = 0.0063 for the number of Iba-1^+^/CD68^+^ microglia; *p* = 0.0007, *p* < 0.0001 and *p* = 0.0490 for the proportion of Iba-1^+^/CD68^+^ microglia) (Figures 4D–F), indicating that microglia were activated in the METH acute withdrawal period. Furthermore, relative to the METH group, the number of Iba-1^+^/CD68^+^ microglia and proportion of Iba-1^+^/CD68^+^ microglia in the Iba-1^+^ cells in the CA1 and DG were significantly reduced in the METH + running group (*p* = 0.0058 and *p* = 0.0090 for the number of Iba-1^+^/CD68^+^ microglia; *p* = 0.0120 and *p* = 0.0457 for the proportion of Iba-1^+^/CD68^+^ microglia) (Figures 4D,F). However, in the CA23 subregion of the hippocampus, treadmill exercise had no obvious effect on the number of Iba-1^+^ cells and Iba-1^+^/CD68^+^ microglia (*p* > 0.05) but significantly reduced the proportion of Iba-1^+^/CD68^+^ microglia in the Iba-1^+^ cells of METH-exposed mice (*p* < 0.0001) (Figure 4E). In addition, we scored the morphology of microglia in the hippocampus (Figure 5E) and the results showed that the percentage of phagocytic phenotype of microglia in the METH group was significantly higher than that in the control group, and treadmill exercise could reverse this change (Figures 5A,C). Additionally, we provided a low magnification image (Iba-1^+^/CD68^+^) of the hippocampus in Supplementary Figure S2. Since astrocytes play an important role in mediating microglia activation, we examined the function of astrocytes in the hippocampus. The results demonstrated that METH exposure could promote the proliferation of astrocytes, and treadmill exercise could reverse this change (Supplementary Figures S3A,B,E). To check the relationship between neuronal death and microglia activity, we also checked the neuronal death in the hippocampus of this model with NeuN immunofluorescence staining. The results indicated that METH exposure reduced the number of neurons in the hippocampus, and treadmill exercise could reverse this change (Supplementary Figures S3C,D,F).

### Treadmill exercise reversed the increased levels of inflammatory cytokines in the striatum and hippocampus of methamphetamine acute withdrawal mice

**FIGURE 6:**
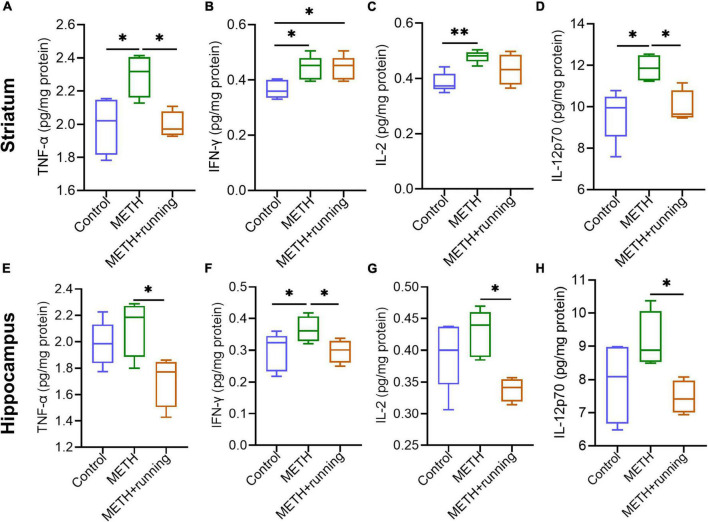
Treadmill exercise counteracted the increase in proinflammatory cytokine levels in the striatum and hippocampus of METH acute withdrawal mice. **(A–H)** Treadmill exercise counteracted the increase in proinflammatory cytokine levels in the striatum **(A–D)** and hippocampus **(E–H)** of METH acute withdrawal mice. The data are expressed as the mean ± SEM. Significance is shown as *p < 0.05, **p < 0.01, ***p < 0.001, and ****p < 0.0001.

To explore the effect of treadmill exercise on the levels of cytokines in the mouse brain, we detected the levels of several classical cytokines in the striatum and hippocampus of mice by the Luminex technique (Figure 6). There were significant differences in the levels of the proinflammatory cytokines TNF-α, IFN-γ, IL-2, and IL-12p70 in the striatum among the three groups (*p* < 0.05). Specifically, compared with the control group, the levels of TNF-α, IFN-γ, IL-2, and IL-12p70 in the striatum were enhanced in the METH group (*p* = 0.0296, *p* = 0.0275, *p* = 0.0070, and *p* = 0.0138, respectively) (Figures 6A–D). Compared with the METH group, the levels of TNF-α and IL-12p70 were significantly diminished in the striatum of mice in the METH + running group (*p* = 0.0292 and *p* = 0.0452, respectively) (Figures 6A,D). However, there were no significant differences in the levels of the cytokines IFN-γ and IL-2 in the striatum of mice in the METH + running group compared with the METH group (*p* > 0.05) (Figures 6B,C). In addition, there were no significant differences in the levels of the cytokines IL-6, IL-5, CXCL1, IL-4, and IL-10 in the striatum of mice among the three groups (Supplementary Figures S4A–E).

Compared with the control group, the IFN-γ level was elevated dramatically in the hippocampus of mice in the METH group (*p* < 0.05) (Figure 6F). However, there were no significant differences in the levels of the cytokines TNF-α, IL-2, IL-12p70, and IL-6 in the hippocampus of mice in the METH group compared with those in the control group (*p* > 0.05) (Figures 6E,G,H and Supplementary Figure S4F). Moreover, compared with the METH group, the levels of the proinflammatory cytokines TNF-α, IFN-γ, IL-2, and IL-12p70 in the hippocampus of mice were significantly decreased in the METH + running group (*p* < 0.05) (Figures 6E–H). In addition, the level of IL-6 in the hippocampus of mice in the METH + running group was prominently increased compared with that in the control group (*p* < 0.05) (Supplementary Figure S4F). However, there were no significant differences in the levels of IL-5, IL-1β, CXCL1, IL-4, and IL-10 in the hippocampus of mice among the three groups (*p* > 0.05) (Supplementary Figures S4G–K).

## Discussion

Anxiety is a common symptom during the acute withdrawal of METH and is an important factor leading to relapse of METH (Zorick et al., 2010; Zhao et al., 2020). In the current study, we constructed a mouse model of anxiety-like symptoms induced by METH acute withdrawal. We set 48 h of METH withdrawal as the time point of this model (acute withdrawal period), which is based on the results of previous population studies, that anxiety symptoms are most obvious in the METH acute withdrawal period (1–2 weeks) (Zorick et al., 2010; Su et al., 2017). In addition, an animal study also demonstrated that rats developed anxiety-like symptoms 48 h after METH withdrawal (Dias et al., 2019). We found that 2 weeks of METH exposure significantly reduced the body weight of mice, which is consistent with a previous study (Lloyd et al., 2017). Acute METH withdrawal decreased the total distance, center entries and center time of mice in the OFT. Moreover, acute METH withdrawal also decreased the percentage of open arm entries and open arm dwell time of mice in the EPM experiment. The above results indicated that the mouse model of acute METH withdrawal–induced anxiety-like symptoms was successfully established.

Previous clinical studies have shown that METH can lead to a metabolic imbalance in METH users, especially the perturbation of energy metabolism and amino acid metabolism (Kim et al., 2020; Wang et al., 2021). To explore the behavioral and metabolic changes that occurred in mice during METH withdrawal, the High-Resolution Behavioral and Metabolic Phenotyping System for Mice was used to monitor the respiration, metabolism and behavior of mice. The results showed that mice in both groups were more active frequently in the dark than in the light, and there was no significant difference in the average locomotor activity between the two groups in the dark and light. Furthermore, the average daily food intake and respiratory exchange ratio in the two groups were higher in the dark than in the light. These results indicate that METH withdrawal did not affect the circadian rhythm or locomotor activity of mice. The results of GLM analysis showed that body mass and activity could affect the energy expenditure, food consumption, oxygen consumption and carbon dioxide production of mice. The higher the body mass and average total activity are, the greater the energy expenditure, food consumption, oxygen consumption and carbon dioxide production. In addition, when the activity of mice was the same, the energy consumption, oxygen consumption and carbon dioxide production of mice in the METH group were higher than those in the control group, but there was no difference in food consumption between the two groups, which suggests that METH withdrawal could affect the energy metabolism of mice. Mitochondria are the site of oxidative metabolism in eukaryotes and the place where sugars, fats and amino acids finally oxidize and release energy. One study found METH-induced striatal mitochondrial dysfunction via oxidative damage in rats (Bazylianska et al., 2021). Another study also proved that short-term methamphetamine withdrawal (12 and 24 h after withdrawal) resulted in metabolic alterations in rat plasma, including amino acid metabolism, energy metabolism and lipid metabolism (Kim et al., 2019). The metabolic alterations induced by METH withdrawal may be associated with psychiatric symptoms (Ma et al., 2021), which need further investigation. An increasing amount of evidence discovered that energy metabolism is associated with anxiety symptoms (Sweeney et al., 2017). High anxiety individuals usually have mitochondrial and metabolic changes, and patients with mitochondrial diseases often suffer from anxiety (Filiou and Sandi, 2019). Additionally, energy metabolism can also regulate central and peripheral inflammatory responses (Maldonado-Ruiz et al., 2017; Boura-Halfon et al., 2019). In this study, METH-induced changes in energy metabolism in mice may be involved in the regulation of microglia activation and central inflammatory response. Moreover, METH may also damage mitochondrial function, alter the energy metabolism of mice, and induce anxiety-like symptoms. The use and withdrawal of METH can cause dysfunction of the peripheral immune system (Loftis et al., 2011), and the alterations of some cytokines in the circulation are significantly related to METH-induced anxiety, depression and cognitive impairment (Huckans et al., 2015; Yang X. et al., 2020). Notably, the METH-induced changes in peripheral immune factors also had remarkable brain-region-specific changes in some cytokines and chemokines (Loftis et al., 2011). This suggests that immune dysfunction may in part contribute to the neuropsychiatric diseases induced by METH. In the current study, the clinical data suggested that most proinflammatory cytokines, including TNF-α, IL-6, IL-12p70, and IL-7, increased significantly and the inflammatory response increased during acute withdrawal of METH, which was consistent with the results of a previous clinical study (Luo et al., 2022). In addition, other cytokines in the circulation also showed significant changes, suggesting immune system dysfunction in METH users during acute withdrawal. We also demonstrated in mice that METH induced anxiety-like symptoms during acute withdrawal and significantly increased the level of the serum proinflammatory cytokine TNF-α, while exercise was able to resist the METH-induced increase in serum TNF-α levels and improve anxiety symptoms. Furthermore, METH also significantly elevated the level of TNF-α in the striatal brain region of mice, and exercise prevented this increase, which indicated that the changes in peripheral TNF-α induced by METH also have specific changes in related brain regions.

Increasing research evidence suggests that neuroinflammation is involved in the occurrence and development of anxiety, depression and neurodegenerative diseases (Yang and Zhou, 2019; Zheng et al., 2021), and the activation of microglia may be related to anxiety (Canedo et al., 2021). Previous studies in rodents have demonstrated that METH is neurotoxic to dopaminergic and serotonergic axons in the striatum, which is associated with microglial activation (LaVoie et al., 2004). Microglia play an important role in immune monitoring, clearing cell debris, synaptic pruning and so on (Felger, 2018; Wang et al., 2018). The striatum is involved in controlling motor activities, learning and memory, anxiety and depression, and reward (Dias et al., 2019; Shah et al., 2021). One clinical study found that acute or chronic METH abuse could cause brain changes at the structural, biochemical and metabolic levels, especially in the striatum (Chang et al., 2007). Our results found that the density of Iba-1^+^/CD68^+^ microglia (activated microglia) in the striatum in METH acute withdrawal mice was higher than that in the control group, demonstrating that METH induced the activation of microglia in the striatum. Many studies have also proven that METH exposure can induce microglial activation in the striatum (Lloyd et al., 2017; Nakagawasai et al., 2022). Kays and Yamamoto (2019) evaluated the mRNA expression of microglia from the rat striatum and prefrontal cortex after METH withdrawal for 2 h or 3 days. Their results showed that these genes associated with microglial activation were significantly upregulated in the striatum and prefrontal cortex 2 h after METH withdrawal; however, after 3 days of METH withdrawal, most of the upregulated genes returned to normal levels (Kays and Yamamoto, 2019). Moreover, Thomas et al. (2004) found that the maximum increase in microglial activation in the mouse striatum induced by METH occurred at 24 and 48 h after METH withdrawal and returned to normal levels at 7 days after METH withdrawal (Thomas et al., 2004). Our study demonstrated that microglia were prominently activated in the mouse striatum 48 h after METH withdrawal. This finding indicates that microglial activation in the striatum is related to the duration of METH withdrawal. In addition to the activation of striatal microglia, the simultaneous increase in proinflammatory cytokine levels also plays crucial roles in the development of anxiety-like symptoms (Kim and Jeon, 2018). In the present study, we found that the levels of the proinflammatory cytokines TNF-α, IFN-γ, IL-2, and IL-12p70 in the mouse striatum were significantly elevated during acute METH withdrawal, and treadmill exercise inhibited the increase in TNF-α and IL-12p70 levels. Another study also reported that METH exposure increased the proinflammatory IL-1β and IL-18 levels in the mouse striatum (Du et al., 2017). This suggests that treadmill exercise can reduce the neuroinflammation of the mouse striatum induced by METH.

The hippocampus can modulate emotional states, especially anxiety states (Bannerman et al., 2014). In the current study, we found that the number of Iba-1^+^ cells was prominently increased in the mouse CA1 and CA23 regions but not in the DG region during acute METH withdrawal compared with the control group, which may be due to the different sensitivities of hippocampal subregions to external stress. Moreover, the number of Iba-1^+^/CD68^+^ microglia and their percentage in Iba-1^+^ cells were significantly elevated in the mouse CA1, CA23, and DG regions during acute METH withdrawal compared with the control group, which is consistent with the study by Lwin et al. (2021), which showed that repeated METH administration induced microglial activation in the mouse hippocampus. Another study based on human samples also suggested that chronic METH exposure induced Iba-1 upregulation in the CA1 region of the postmortem hippocampus (Mahmoudiasl et al., 2019). In addition, we found that the levels of IFN-γ increased obviously in the hippocampus during acute METH withdrawal. However, there were no significant differences in the levels of the proinflammatory cytokines TNF-α, IL-6, IL-12p70 and IL-1β in the mouse hippocampus between the METH group and the control group, which is inconsistent with the results of two other studies by Liskiewicz et al. (2019) and Namyen et al. (2020). Liskiewicz et al. (2019) proved that METH administration increased the levels of IL-1β in the rat hippocampus, and Namyen et al. (2020) also indicated that METH prominently elevated the levels of IL-1β, IL-6 and TNF-α in the rat hippocampus compared to the control group. Different kinds of experimental animals and different METH administrations may be the main reasons for the different results. It is worth mentioning that both RANTES/CCL5 and eotaxin/CCL11 are reported to be associated with METH abuse (Najera et al., 2016; Huckans et al., 2017), and CCR5 is used as a therapeutic target for brain injury (Joy et al., 2019). In this study, the levels of RANTES/CCL5 and eotaxin/CCL11 in peripheral plasma of METH users were also significantly increased than those of healthy controls, indicating that they were significantly related to METH abuse and may be an important target for immunotherapy intervention.

Accumulating evidence has illustrated the importance of microglial activation and proinflammatory cytokines in METH-induced neurotoxicity and anxiety-like symptoms. One study found that METH-induced decreases in dopamine and serotonin and reactive gliosis were attenuated in IL-6 knockout mice (Ladenheim et al., 2000). A recent study demonstrated that METH activates microglia dependent on astrocyte-derived TNF and glutamate (Canedo et al., 2021). It was also observed that anxiety-like behavior was attenuated by minocycline processing anti-inflammatory properties by targeting microglia in the DG of the mouse hippocampus (Rooney et al., 2020). Under normal conditions, microglia are resting, in the presence of endogenous or exogenous pathological injury, microglia surface receptors recognize pathogens, cellular debris or abnormal proteins and induce microglia activation (Leng and Edison, 2021). Activated microglia are phagocytic and produce large amounts of harmful inflammatory factors that interfere with normal neural signaling and induce the loss of dopaminergic neurons and neuronal apoptosis, which result in motor dysfunction, cognitive decline and emotional disorders (Gordon et al., 2018; Yao et al., 2019; Guo et al., 2022). In this model, after METH exposure, microglia in hippocampus and striatum were significantly activated, and proinflammatory cytokines in these two brain regions were significantly increased, which may affect the normal transmission of neural signals, leading to anxiety symptoms. Several studies found that METH induced microglia activation was related to neurotoxicity (LaVoie et al., 2004; Thomas et al., 2004). However, some study groups claim that microglial activity is not related to neurotoxicity in METH administration (Shaerzadeh et al., 2018; Sabrini et al., 2020). This controversial result may be related to the different dosage and time of METH use in different animal models. In our model, we discovered that METH exposure reduced the number of neurons in the hippocampus, and treadmill exercise could reverse this change, indicating that the activation of microglia is related to neurotoxicity. At present, some new evidence shows that METH activated astrocytes to express complement C3 (He et al., 2022), and astrocytic C3- microglial C3aR is a critical signal pathway highly related to neuronal injury (Chen et al., 2020; Wei et al., 2021). Furthermore, the complement C3- C3aR also related to anxiety behavior (Westacott et al., 2022). Our study discovered that METH can activate astrocytes and microglia, and the activated astrocytes and microglia may mediate synaptic loss and neuronal damage through astrocytic C3- microglial C3aR pathway, thus inducing anxiety-like symptoms, which needs to be verified by following studies. METH can act on neurons or glial cells to release proinflammatory molecules; at the same time, circulating immune factors induced by METH may also enter the central nervous system through the damaged blood–brain barrier. These proinflammatory molecules might further stimulate the downstream neuronal apoptosis signaling pathway, leading to neuronal cell death and/or glial activation, which further aggravates neurotoxicity and neuroinflammation (Park et al., 2017). Therefore, it is important to inhibit METH-induced peripheral and central inflammation. Proper exercise is an effective therapeutic strategy for METH addiction and withdrawal syndrome (Huang et al., 2019; He et al., 2021); however, there are few studies on the mechanism of exercise detoxification. It has not been reported that treadmill exercise can improve anxiety-like symptoms in METH acute withdrawal by counteracting the central inflammation induced by METH. Consistent with the results of a previous clinical study (Yang J. et al., 2020), our behavioral results demonstrated that moderate-intensity treadmill exercise can alleviate anxiety-like symptoms during acute METH withdrawal in mice. Numerous studies in related fields have shown that exercise can regulate microglial activation and the inflammatory response in neurodegenerative diseases, such as Alzheimer’s disease (Lu et al., 2017). However, it is unclear whether moderate-intensity treadmill exercise will have beneficial effects on microglia in the mouse striatum and hippocampus during acute METH withdrawal. In the current study, we found that treadmill exercise attenuated the increase in the number of Iba-1^+^/CD68^+^ microglia and their percentage in Iba-1^+^ cells induced by acute METH withdrawal in the striatum, CA1 and DG regions of the hippocampus. However, treadmill exercise did not attenuate the increase in Iba-1^+^ cells induced by METH in the CA1 and CA23 subregions. This finding suggests that treadmill exercise mainly affects the functional state of microglia in the CA1 and CA23 of the mouse hippocampus but has no effect on the number of microglia. These results indicated that moderate-intensity treadmill exercise can counteract the activation of microglia in the mouse striatum and hippocampus induced by METH. Previous studies in the field of neurodegenerative diseases have also demonstrated that treadmill exercise could inhibit microglial activation in mouse and rat hippocampi (Mee-Inta et al., 2019).

Considerable evidence has demonstrated that exercise can reduce microglial activation by downregulating the levels of proinflammatory molecules (Mee-Inta et al., 2019). TNF-α is a major proinflammatory cytokine in the central nervous system. A clinical study showed that antitumor necrosis factor (anti-TNF) could decrease anxiety in inflammatory bowel disease patients (Siebenhuner et al., 2021). Other studies in rodents have also demonstrated that TNF-α is closely related to the occurrence of anxiety symptoms (Alshammari et al., 2020; Dib et al., 2021). Our results found that treadmill exercise reduced the level of TNF-α in the striatum, hippocampus and serum of the METH acute withdrawal mice, which may become the main reason for treadmill exercise alleviating anxiety-like symptoms in the METH acute withdrawal period. In addition, our study found that treadmill exercise significantly decreased the levels of IL-12p70 in the striatum and hippocampus of mice with acute METH withdrawal, indicating that IL-12p70 may play an important role in acute METH withdrawal–induced anxiety-like symptoms, which needs further research. Furthermore, in the present study, treadmill exercise also reduced the levels of IFN-γ and IL-2 in the hippocampus of METH acute withdrawal mice. The above results illustrated that moderate-intensity treadmill exercise exerts a protective effect by counteracting the increase in some proinflammatory cytokines in the striatum, hippocampus and serum of METH acute withdrawal mice. In summary, we infer that the anti-anxiety effect of treadmill exercise in this model may be achieved by inhibiting microglial activation and the release of proinflammatory cytokines. Accumulative evidence showed that METH can act on TLR4 receptor on microglia and induce NF-κB activation, thereby increasing the production of proinflammatory cytokines (Lwin et al., 2021). Treadmill exercise may reduce METH induced microglia activation and proinflammatory cytokine production by regulating TLR4/NF-κB pathway, which needs further research.

As we all know, exercise is beneficial to the treatment of many neurodegenerative diseases and mood disorders. Accumulative studies showed that exercise can improve brain function in many ways such as neurotransmitter release, neurotrophic factors and neurogenesis, and changes in cerebral blood flow (Bherer, 2015; Mikkelsen et al., 2017). METH can induce blood-brain barrier damage and oxidative stress, which lead to behavioral abnormality in the mice (Huang et al., 2021). Physical exercise has been shown to ameliorate the oxidative stress in the brain and exert the neural protective effect (Malkiewicz et al., 2019). Therefore, in our model, treadmill exercise may also ameliorate anxiety-like symptoms by resisting METH induced oxidative stress response. Furthermore, METH also can increase tau phosphorylation and APP processing (Panmak et al., 2021), which may be responsible for the development of the anxiety-like symptoms in this model. And treadmill exercise may ameliorate anxiety-like symptoms by regulating tau and APP.

## Conclusion

Our study illustrated that acute withdrawal of METH leads to peripheral immune dysfunction. METH exposure induced anxiety-like symptoms in the acute withdrawal period and resulted in an imbalance of energy metabolism in model mice. In addition, the activation of microglia and neuroinflammation in the striatum and hippocampus may be involved in the pathogenesis of METH acute withdrawal-induced anxiety-like symptoms in model mice, and treadmill exercise could attenuate these changes. It should be noted that there is a lack of female METH users and female mice in our study, and the results are only applicable to male METH users and male mice. Our report provides new clues for the immunomodulatory target of exercise against acute METH withdrawal–induced anxiety symptoms, which will provide a reference for future scientific research and clinical treatment.

## Data availability statement

The datasets presented in this study can be found in online repositories. The names of the repository/repositories and accession number(s) can be found below: the datasets for this study can be found in the Mendeley Data. The reserved DOI is: 10.17632/7zbggkpmdy.1.

## Ethics statement

The studies involving human participants were reviewed and approved by the Research Ethics Committee of the First Affiliated Hospital of Kunming Medical University (No. 2018 Lunshen L42). The patients/participants provided their written informed consent to participate in this study. The animal study was reviewed and approved by the Animal Experiment Ethics Review Committee of Kunming Medical University (Approval number: kmmu20211261).

## Author contributions

G-FR, K-HW, and Y-QK designed the experiments, developed the methodology, analyzed and interpreted the data, and wrote the manuscript. G-FR, XW, RZ, ZZ, and YL performed the experiments and acquired the data. HL, J-QY, DK, and HYL provided technical support and supported data analysis and interpretation. K-HW and Y-QK supervised the study and were responsible for coordination and strategy. All authors have approved the final manuscript for publication.
