# Supplementary material for: Exercise modulates central and peripheral inflammatory responses and ameliorates methamphetamine-induced anxiety-like symptoms in mice
Source: Front Mol Neurosci. 2022 Aug 29;15:955799. doi: 10.3389/fnmol.2022.955799 (PMC9465459; doi:10.3389/fnmol.2022.955799)
Supplement: Supplementary file 1 [file Data_Sheet_1.docx]

Supplementary Material

## Supplementary Tables

**Table S1.** Alteration of serum cytokine levels in METH acute withdrawal versus control

| **Cytokines** | **Control** | **METH (AW)** | **^a^*p value*** |
| --- | --- | --- | --- |
| IL-5 | 12.17 ± 3.18 | 12.43 ± 9.20 | 0.9004 |
| IL-4 | 3.89 ± 1.32 | 4.74 ± 4.34 | 0.1683 |
| IL-8 | 26.21 ± 12.98 | 35.22 ± 23.86 | 0.1206 |
| IL-10 | 5.32 ± 2.69 | 17.24 ± 22.48 | 0.2798 |
| IL-17A | 16.16 ± 7.772 | 14.65 ± 6.41 | 0.4820 |
| VEGF | 532.5 ± 498.4 | 1370 ± 1540 | 0.2148 |
| MCP-1 | 77.08 ± 30.81 | 94.39 ± 77.73 | 0.3277 |

The data were expressed as mean ± standard deviation.

Abbreviations: AW, acute withdrawal.

^a^Analyzed by the Unpaired t test or Mann Whitney test.

*P* < 0.05 was considered as a significant difference.

**Supplementary Figures**

**
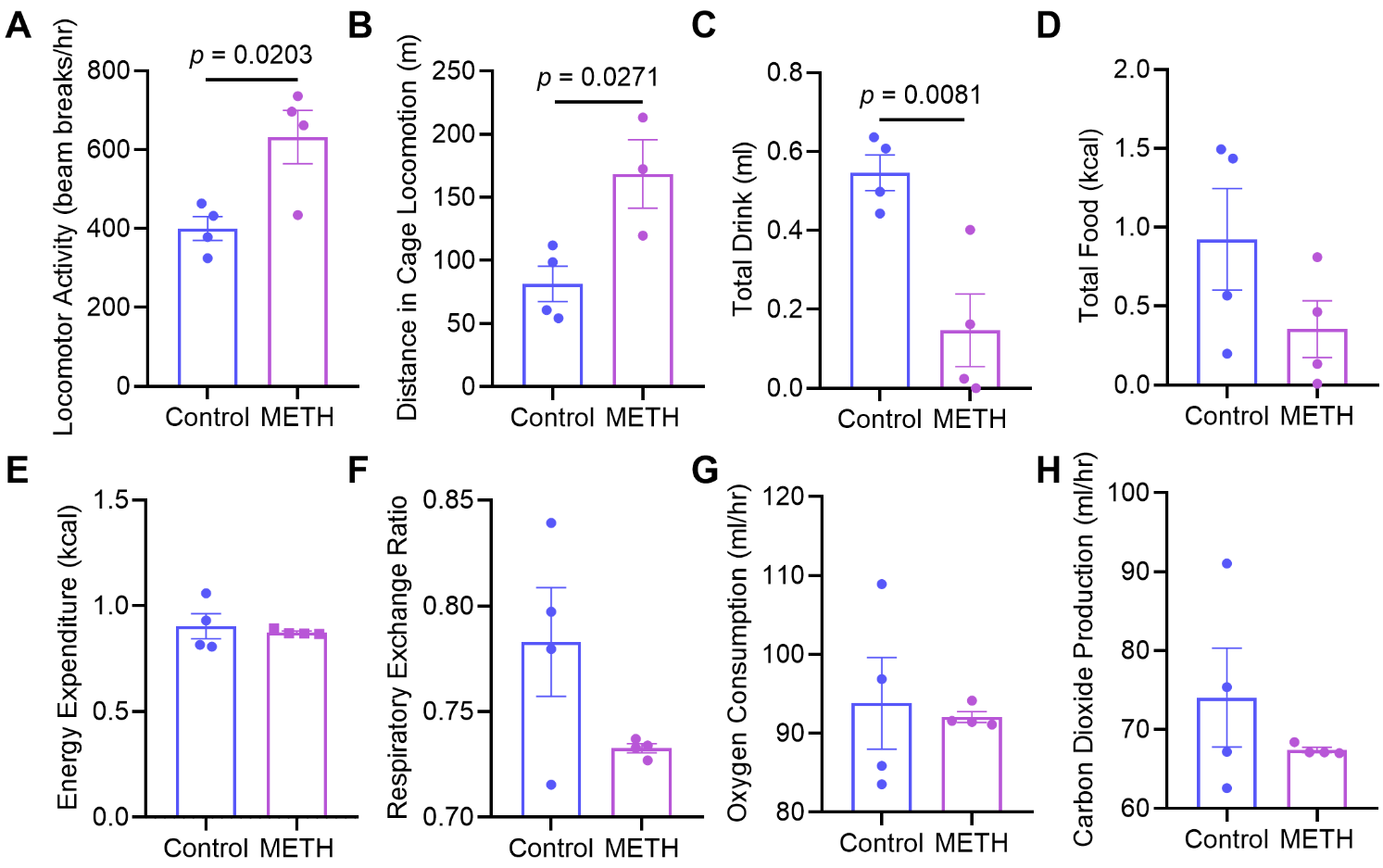
**

**Figure S1. The acute behavior and physiologic measurements in mice within 3 hours after intraperitoneal injection of METH. A** The locomotor activity of mice within 3 hours in the two groups after intraperitoneal injection of METH or saline. **B** The total movement distance of mice within 3 hours in the two groups after intraperitoneal injection of METH or saline. **C-H** Changes in metabolic parameters (total drink, total food, energy expenditure, respiratory exchange ratio, oxygen consumption and carbon dioxide production) in the METH group (n = 4) and control group (n = 4). The data were compared with an unpaired *t* test and are expressed as the mean ± SEM, and p < 0.05 was considered a significant difference.


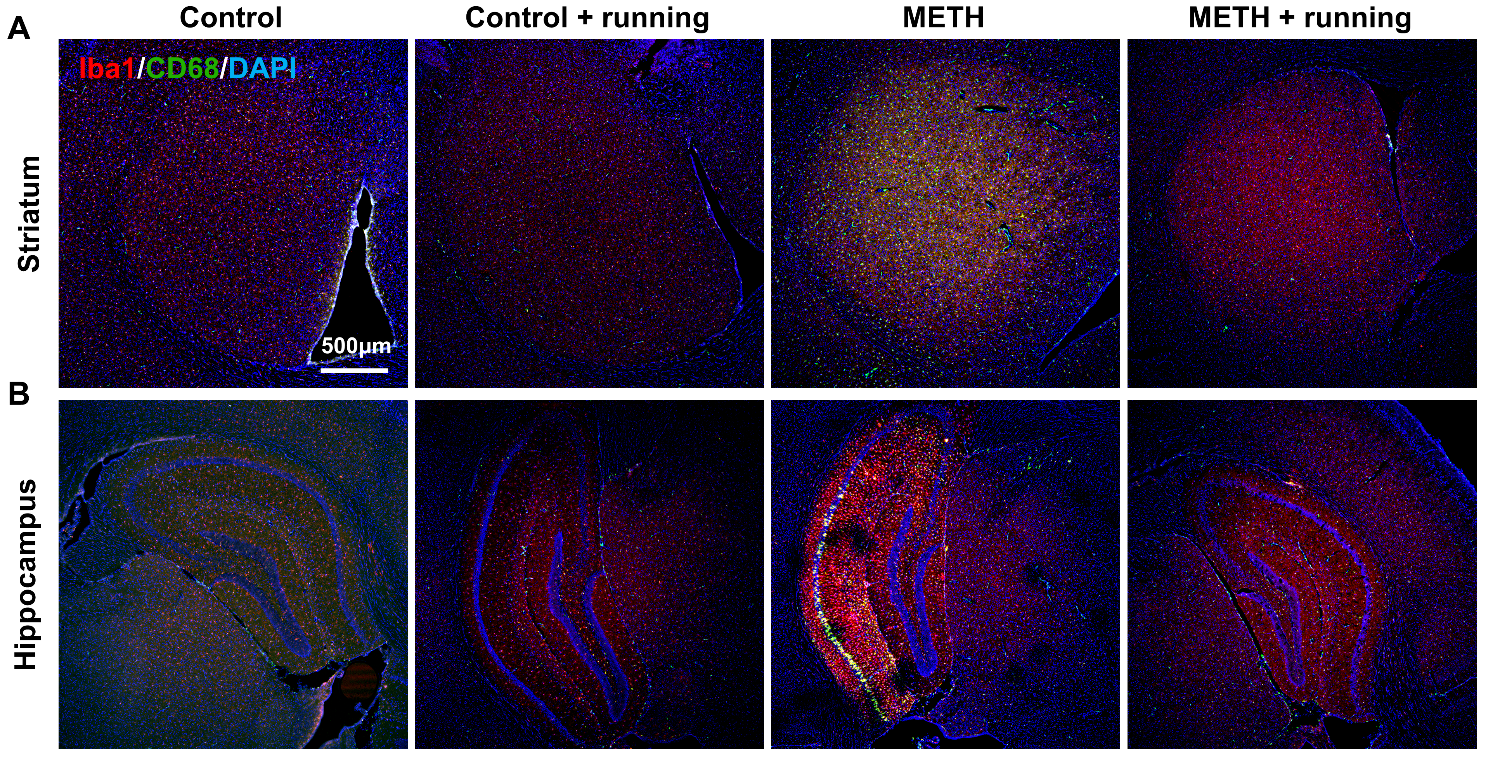


**Figure S2. The low magnification image of activated microglia (Iba1/CD68) in the striatum and hippocampus.** A Immunofluorescence staining of striatal sections from four groups of mice. The red represents Iba1, the green represents CD68, and the blue represents DAPI. **B** Representative images of immunofluorescence staining (Iba1/CD68) in the hippocampus of four groups of mice. Representative images of n = 3 mice per group.


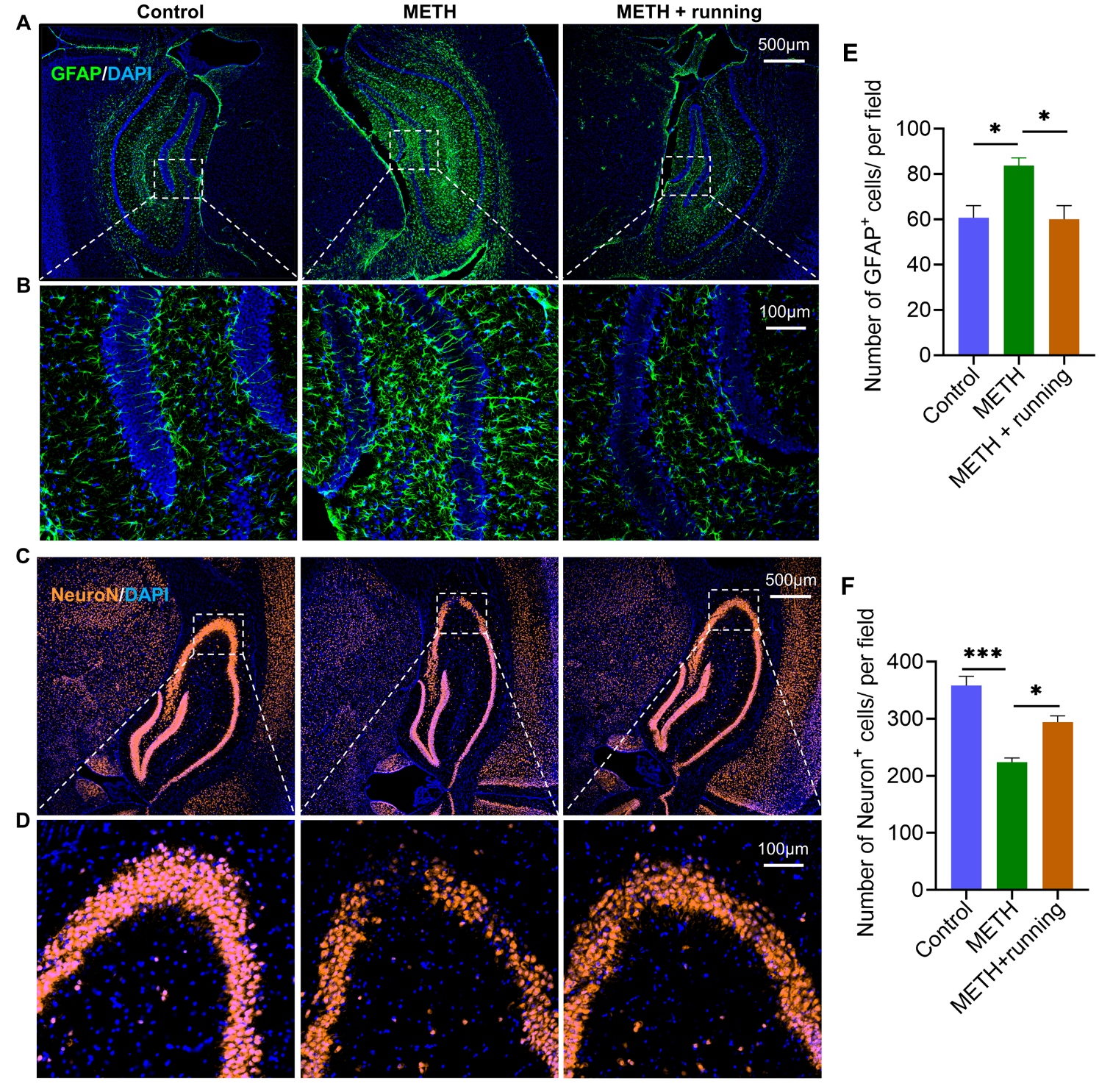


**Figure S3. Treadmill exercise reduced astrocyte proliferation in the hippocampus of mice with acute METH withdrawal. A** Representative low magnification image of astrocytes (GFAP) in the hippocampus of the three groups. **B** The high magnification image of astrocytes (GFAP) in the hippocampus of the three groups. **C** Representative low magnification image of neurons (NeuroN) in the hippocampus of the three groups. **D** The high magnification image of neurons (NeuroN) in the hippocampus of the three groups. **E** The number of astrocytes in the hippocampus. **F** The number of neurons in the hippocampus. Representative images of n = 3 mice per group. All results represent the average of three indep**e**ndent experiments. The data are expressed as the mean ± SEM. Significance is shown as *p <​ 0.05, **p <​ 0.01, ***p <​ 0.001, and ****p <​ 0.0001.


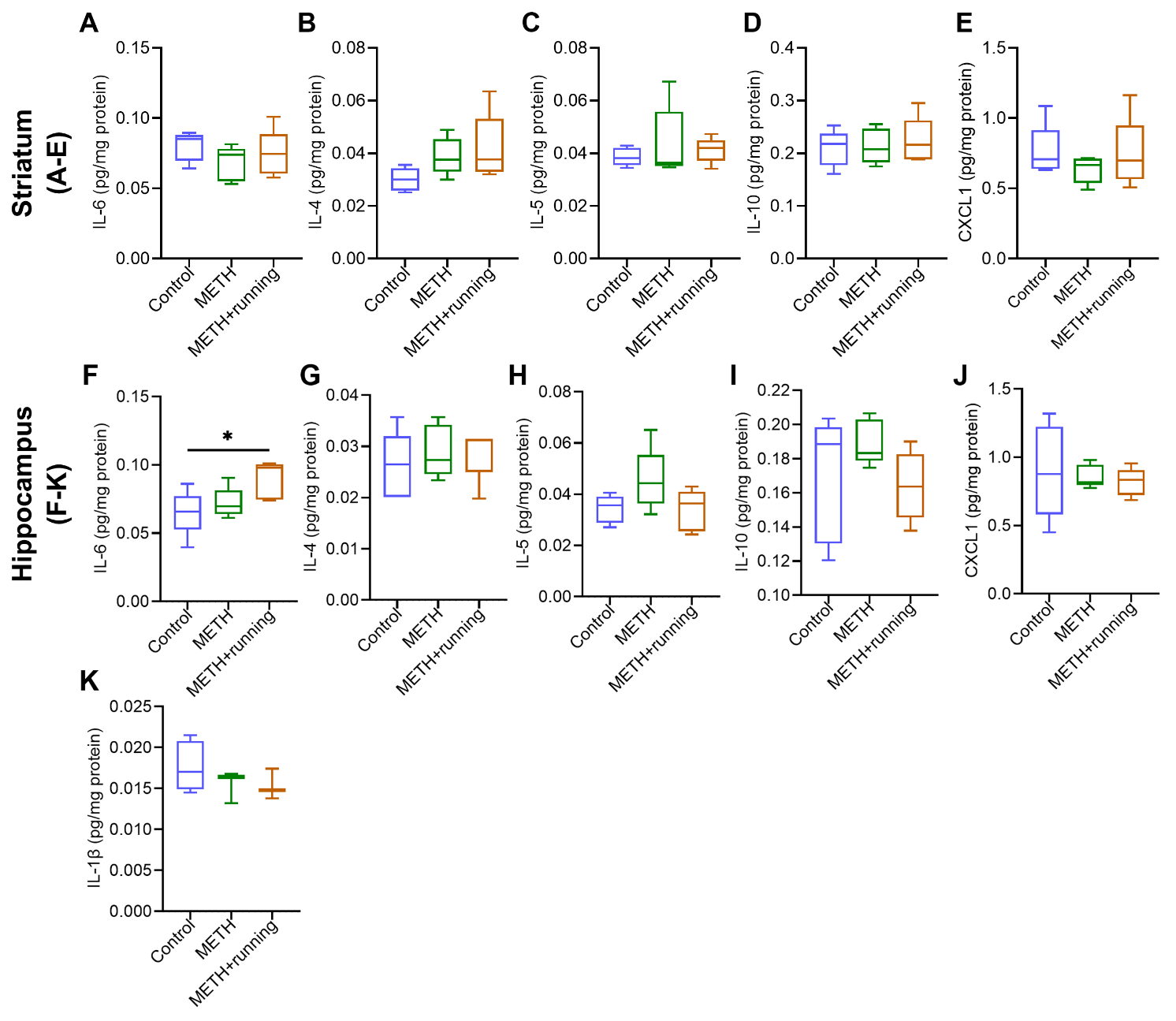


**Figure S4. The cytokine levels in the hippocampus and striatum of three groups of mice. A-E** The cytokine levels in the striatum of three groups of mice. These cytokines including IL-6 (**A**), IL-4 (**B**), IL-5 (**C**), IL-10 (**D**) and CXCL1(**E**). K-F The cytokine levels in the hippocampus of three groups of mice. These cytokines including IL-6 (**F**), IL-4 (**G**), IL-5 (**H**), IL-10 (**I**), CXCL1(**E**) and IL-1β (**K**). The data are expressed as the mean ± SEM. Significance is shown as *p <​ 0.05, **p <​ 0.01, ***p <​ 0.001, and ****p <​ 0.0001.
